# Outcomes of a cementless onlay short stem reverse shoulder arthroplasty in elderly patients: a comprehensive analysis of clinical and radiological findings

**DOI:** 10.1007/s00402-024-05321-6

**Published:** 2024-04-23

**Authors:** Rui Claro, Arnaldo Sousa, Eurico Silva, Luís Henrique Barros

**Affiliations:** 1 Department of Orthopedics, Centro Hospitalar Universitário de Santo António. Hospital de Santo António, Porto, Portugal; 2 Shoulder Unit, Department of Orthopedics, Centro Hospitalar Universitário de Santo António. Hospital de Santo António, Porto, Portugal; 3 Instituto de Ciências Biomédicas Abel Salazar da Universidade do Porto (ICBAS-UP), Porto, Portugal

**Keywords:** Elderly, Humeral Stem, Reverse Shoulder Arthroplasty, Shoulder Joint, Shoulder prosthesis, Short stem.

## Abstract

**Objective:**

The objective of this study was to evaluate clinical and radiological outcomes of a short stem reverse shoulder prosthesis with metaphyseal fixation specifically in older patients.

**Methods:**

All patients, older than 70 years, submitted to a Reverse Shoulder Arthroplasty (RSA) using a cementless onlay short stem (Aequalis Ascend™ Flex Convertible Shoulder System (Stryker®)) from January 2017 to December 2021, with a minimum follow-up of 2 years, were included. Postoperative radiographs were assessed for stem loosening, subsidence, and varus-valgus tilt. Range of motion, visual analogue scale for pain, constant score, complication rate and revision rate were also analysed.

**Results:**

A total of 34 patients with a mean age of 75 years (range 71–83 years) were submitted to a cementless onlay short stem RSA with a Bone Increased Off-Set (BIO-RSA) construct. The mean follow-up period was 61 months (range 54–87). Significant improvements (*p* < 0.001) were observed for the constant score and range of motion from the preoperative state to final follow-up. One case exhibited a significant varus deviation (> 5˚) during the follow-up period. No case of stem loosening was identified. There was only one case of complication because of post-traumatic dislocation, but the stem didn’t need revision.

**Conclusion:**

Short stem RSA, even in patients older than 70 years, can yield a stable fixation with a good clinical and radiological outcome at short-medium term follow-up.

**Level of Evidence:**

Level III; Retrospective Study

**Supplementary Information:**

The online version contains supplementary material available at 10.1007/s00402-024-05321-6.

## Introduction

Total shoulder arthroplasty (TSA) employing uncemented humeral stems, commonly known as press-fit stems, has become the established therapeutic approach for glenohumeral osteoarthritis [1, 2]. Despite offering satisfactory functional improvements and pain relief, uncemented humeral stems in TSA are linked to issues such as bone resorption, stress shielding, and frequent failures associated with the glenoid component [3, 4]. Aseptic loosening of the humeral implant, although less frequently reported than glenoid component issues, presents a potential long-term problem, with a reported 9% rate of humeral loosening at 20 years [4, 5].

Complications related to humeral long stems include stress shielding, aseptic loosening, operative humeral fractures, and traumatic periprosthetic fractures [6, 7]. Furthermore, the revision of both cemented and uncemented stems poses challenges and is often accompanied by substantial bone loss during stem extraction [8]. Preserving most of the humeral bone stock has emerged as a new objective in total shoulder replacement surgery, particularly in an aging population where revisions are more prevalent [9].

In response to these challenges, short cementless stems with metaphyseal fixation have been introduced to conserve bone stock, reduce stress shielding, eliminate diaphyseal stress risers, and simplify stem removal during revision [8, 10, 11]. Several press-fit short stems with diverse designs are currently available. Among them, the Aequalis Ascend™ Flex Convertible Shoulder System (Stryker®) stands out with its curved shape, proximal porous coating, and convertibility between anatomic TSA and Reverse Shoulder Arthroplasty (RSA).

While earlier investigations involving this stem have shown promise [12–14], there is a paucity of studies specifically examining this prosthesis in an older population.

As the global population continues to age, the prevalence of older patients requiring RSA is expected to rise. Therefore, the objective of this study is to assess the clinical and radiological outcomes of RSA utilizing a humeral short stem with metaphyseal fixation in patients aged 70 years and above.

## Methods

A retrospective review was conducted at a single institution between January 2017 and December 2021 to analyze all consecutive patients aged 70 years and older who underwent RSA with a short stem featuring proximal porous coating, specifically the Aequalis Ascend™ Flex Convertible Shoulder System (Stryker®).

The inclusion criteria comprised primary RSA for patients aged 70 years or older, with a preoperative diagnosis of cuff tear arthropathy, massive rotator cuff tear, failed rotator cuff repair, primary osteoarthritis, or rheumatoid arthritis. A minimum follow-up period of 2 years was mandatory. Patients with fracture sequelae, a history of infection, shoulder neoplasm, prior open shoulder surgery, or presence of neurologic problems, such as Parkinson’s disease or axillary nerve lesion were excluded.

The protocol was approved by the Institutional Review Board of the Centro Hospitalar Universitário de Santo António, and informed consent was obtained from all participants.

### Surgical technique

All surgical procedures were performed by a single shoulder surgeon (RC) using the Aequalis Ascend™ Flex Convertible Shoulder System (Stryker®) with a Bone Increased Offset – Reverse Shoulder Arthroplasty (BIO-RSA) configuration. The surgeries were conducted through a deltopectoral approach with the patient in the beach-chair position.

The subscapularis or its remnants were detached from the lesser tuberosity using a “peeling tenotomy” technique. Whenever feasible, the subscapularis was reattached with nonabsorbable transosseous sutures to ensure tension-free fixation, with the arm positioned neutrally. A tenodesis of the long head of the biceps tendon to the pectoralis major tendon was performed if the long head of the biceps tendon was present.

All humeral implants were uncemented. The final humeral stem diameter corresponded to the number below the last probe used. The retroversion angle was set at 20° by aligning the version rod to the forearm. The humeral implants were applied in an onlay fashion, featuring a reverse tray with 1-mm thickness and 1.5-mm offset at the “6” position, utilizing a polyethylene 36 insert and achieving a final neck-shaft angle of 145°.

The glenoid baseplate was positioned on the inferior margin of the glenoid rim with 10° of glenoid inferior tilt. The BIO-RSA technique involved harvesting a 10-mm-thick cylindrical autograft of cancellous bone from the humeral head, following the approach described by Boileau et al. [15]. A glenoid baseplate implant with a 29-mm diameter and an extended 25-mm central long post was utilized to ensure contact between the graft and the glenoid. A centered 36-mm glenosphere was used in all patients.

Following liner engagement and reduction, the arm underwent stability and tension testing. A drain was utilized in all cases.

Postoperatively, the arm was immobilized in a sling for 4 weeks. Passive mobilization was initiated immediately after the operation. After the initial 4 weeks, the sling was discontinued, and active range of motion exercises were initiated, with patients allowed to resume activities of daily living.

### Clinical and radiologic evaluation

All participants received comprehensive information about the study and provided informed consent before inclusion.

A standardized examination protocol was implemented for patients both preoperatively and during follow-up. Radiographs were taken at various intervals, including preoperative, immediate postoperative, 6 weeks, 6 months, 1 year, and subsequently every 6 months, to assess radiographic changes. Standardized radiographs, including anteroposterior, axillary, and scapular Y views, were obtained. Anteroposterior views were captured with the forearm in a neutral rotation to ensure uniform and comparable images.

Clinical evaluations included the Constant Score (CS), range of motion assessments, and Visual Analog Scale (VAS) for pain (0 indicating no pain and 10 indicating the worst pain). Range of motion measurements encompassed active forward elevation in the scapular plane, abduction, adduction, external rotation, and internal rotation. Strength assessments were conducted using a handheld dynamometer, with the shoulder in neutral rotation and 90° of abduction in the scapular plane. Clinical evaluations were conducted by two orthopedic surgeons (RC and LB) before surgery, at 6 months postoperatively, at 1 year, and at the 2-year follow-up, with a final follow-up clinical assessment for the study.

Postoperative radiographs were analyzed for stem loosening, subsidence, and varus-valgus tilt. Two orthopedic surgeons (AS and LB) conducted the assessments, reaching a consensus in cases of discrepancies.

Bone remodeling and stress shielding were analyzed following the methodologies introduced by Nagels et al. [4] and Schnetzke et al. [16]. The zones around the humeral stem were divided into five regions: lateral proximal, lateral-distal, medial-proximal, medial-distal, and under the tip of the stem. These zones were examined for two features of bone remodeling: (1) cortical thinning and osteopenia (CNO); (2) spot welds (SW) around the complete humeral component. CNO and spot weld findings were categorized as absent (no or minor findings) or present (moderate or severe) to facilitate proper assessment.

Stem loosening and subsidence were defined based on criteria outlined by Sanchez-Sotelo et al. [8]. Humeral component fixation was categorized using the grading system proposed by Sperling et al. [17], and partial or total greater tuberosity resorption was measured.

Stem inclination relative to the humeral shaft axis was measured in degrees, categorized as neutral if the stem angle in relation to the humeral axis was ± 5°, valgus if the angle was > 5°, and varus if the angle was < 5°.

### Statistical analysis

Statistical analysis was conducted using the IBM Statistical Package for Social Sciences (SPSS), version 23. Descriptive statistics were computed, and the normal distribution of data was assessed using the Kolmogorov-Smirnov test. Paired samples t-tests were employed to analyze differences between preoperative and postoperative continuous data.

For categorical variables, Chi-square and Fisher’s exact tests were applied as appropriate. Additionally, t-tests and Mann-Whitney U-tests were utilized to compare radiographic and clinical data among patients, depending on the nature of the variable under consideration.

A significance level of *p* ≤ 0.05 was adopted, and values at or below this threshold considered statistically significant.

## Results

A total of 34 patients, comprising 7 men and 27 women, with an average age of 75 years (range 71–83 years), were identified. The mean follow-up period was 61 months (range 54–87).

Significant improvements were observed in the Constant Score, Visual Analog Scale (VAS) pain, and range of motion from the preoperative state to the final follow-up (*P* < 0.001) (Table 1).

The Constant Score increased from a mean of 24.8 ± 12.7 preoperatively to 68.2 ± 17.8 at the 1-year follow-up and 72.6 ± 16.5 at the final evaluation. Similarly, significant improvements were noted for pain and range of motion, as outlined in Table 1.

**Fig. 1:**
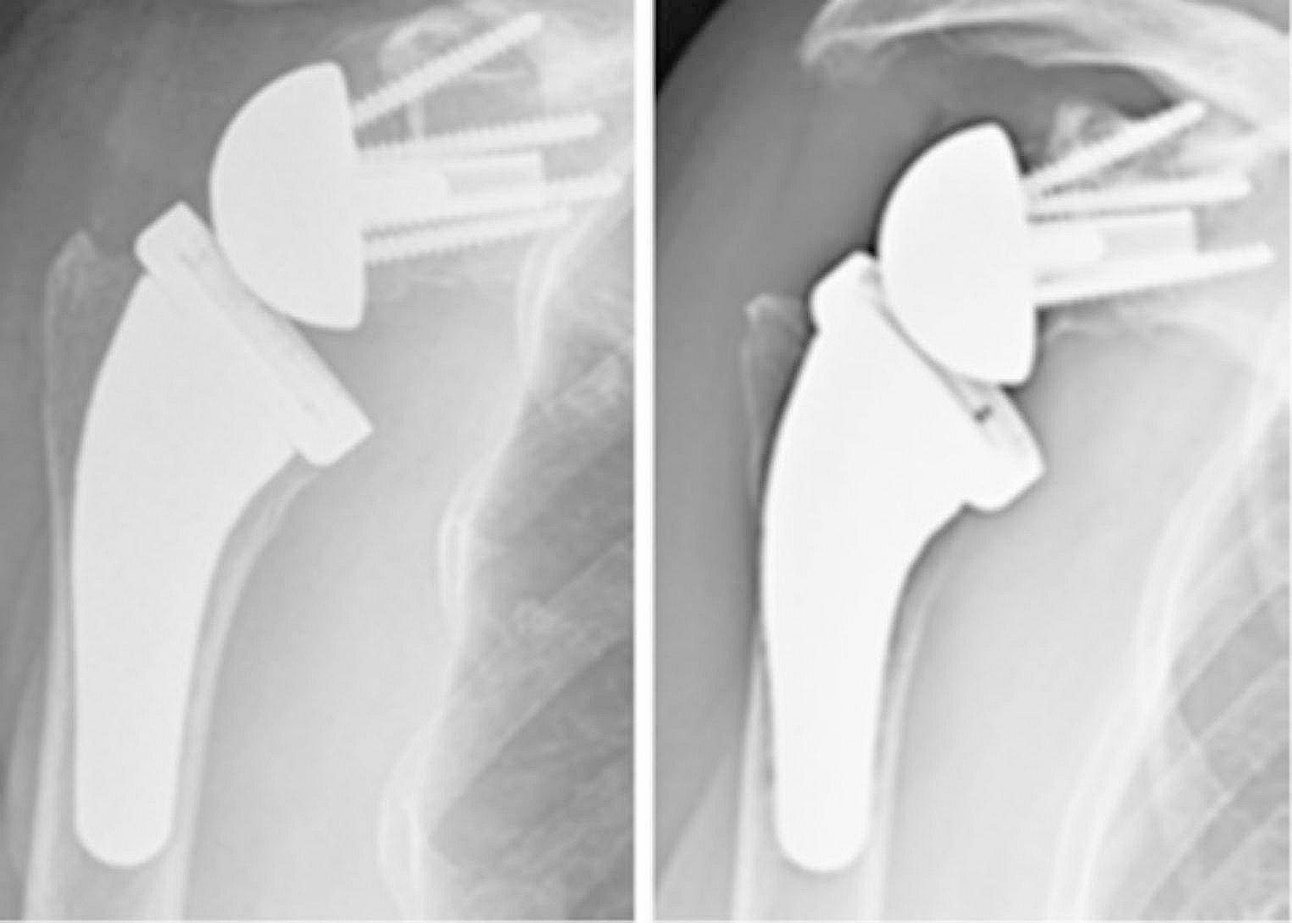
A patient with cortical bone narrowing and osteopenia at the lateral proximal zone and medial proximal zone. Left - immediate postoperative radiograph. Right - at 37 months follow-up

**Fig. 2:**
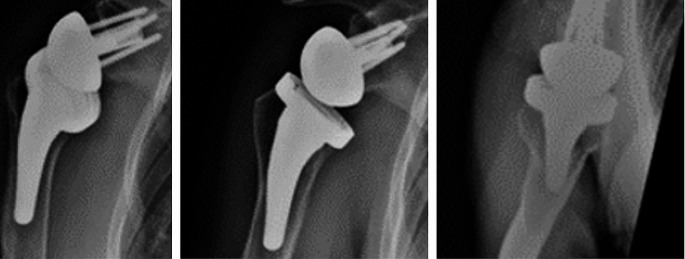
A patient with no stress shielding at the lateral proximal zone and medial proximal zone or spot welds, at 20 months follow-up

Regarding radiographic analysis, no instances of stem subsidence (> 5 mm) were observed, and only one case exhibited a significant varus deviation (> 5˚) during the follow-up period. Importantly, this deviation did not impact the Constant Score (*p* = 0.095). Eight cases (24%) were identified with CNO at the lateral proximal zone and medial proximal zone (Fig. 1). No cases of stem loosening or spot welds were identified (Fig. 2).

**Table 1:** Clinical evaluation preoperatively and at last follow-up

Parameters	Before surgery	1 year follow-up	Last follow-up	*p* value*
Mean Constant Score (pts) ± SD	24.8 ± 12.7	68.2 ± 17.8	72.6 ± 16.5	*< 0.001*
VAS pain	7.2 ± 1.9	1.8 ± 2.3	1.9 ± 2.7	*< 0.001*
Range of Motion (degrees)				
Anterior Flexion	62 ± 23	128 ± 35	129 ± 37	*< 0.001*
Abduction	59 ± 28	117 ± 41	119 ± 43	*< 0.001*
External Rotation	11 ± 21	24 ± 12	23 ± 14	*0.041*

VAS: Visual Analogue Scale for Pain

* Students t test applied. The comparison is made between before surgery and at last follow-up

Only one case experienced a complication, attributed to posttraumatic dislocation, which was successfully treated with closed reduction. No other complications were observed.

## Discussion

This study innovatively explores RSA outcomes in individuals aged over 70, utilizing a cementless onlay short stem prosthesis system (Aequalis Ascend™ Flex Convertible Shoulder System - Stryker®). Notably, significant improvements in the Constant Score (CS), VAS pain, and range of motion were observed from preoperative to final follow-up (*P* < 0.001), culminating in a mean CS of 72.4. These outcomes particularly are noteworthy considering the older age group under investigation.

Primary RSA has become a successful procedure with improvements in pain, motion, function, and high patient satisfaction [18–26]. It is a safe and effective procedure in elderly patients, with a relatively low rate of medical and surgical complications [27–33]. 

The clinical results align with those of previous studies on short stem shoulder prostheses, despite variations in age groups [9, 34–37]. This study bridges a gap in the literature by specifically analyzing the Ascend™ Flex short stem in an older age cohort. For instance, Schnetzke et al. [36] reported on 24 RSA cases employing the same stem as our study, with a mean follow-up of 25 months, and reported an improvement in CS of 22 to 57 from pre-operatively to the final follow-up. Similarly, Casagrande et al. [34] documented results from 69 anatomic TSA cases utilizing first-generation Ascend™ Flex short stem prostheses, showing an improvement in CS from 39 to 68 with a minimum follow-up of 24 months. Additionally, findings from Linke et al. study [38]are consistent with ours, indicating favorable clinical outcomes and low complication rates in primary reverse total shoulder arthroplasty using an cementless humeral short stem.

Radiological findings from studies such as that of Garofalo et al. [39] suggest that RSA with a standard cementless and metaphyseal stem fixation is a viable option for the treatment of complex proximal humeral fractures. These results are consistent with studies employing short cementless humeral stems, like those of Abduh et al. [40], which found no radiographic differences between TSA and RSA. Similarly, study by Larose et al. [41] demonstrated low revision rates and a low prevalence of humeral stems at risk of radiographic loosening with a press-fit short stem humeral design.

Our radiological findings revealed CNO in 24% of cases at the lateral proximal and medial proximal zones, indicative of potential stress shielding. Comparisons with studies by Raiss et al. [14] and Schnetzke et al. [36] show similar radiographic findings, emphasizing the consistency in bone adaptations with the use of short stems.

In Schnetzke et al.‘s study [36], which included 53 shoulder prosthesis (29 anatomic and 24 reverse replacements) with a follow-up period of 25 months, CNO was reported in the lateral and medial proximal zones in 42% and 10% in the distal zones These bone changes were attributed to a higher filling ratio of the stem in the humeral canal.

Kramer et al. [42]conducted a study evaluating the effects of stem length and width on proximal humerus stress shielding in uncemented primary RSA. Their findings suggested that while short and standard stems for RSA yield favorable results after 2 years, higher stem length and width had a significant negative effect on stress shielding. They recommended that short stems with a filling ratio at the metaphyseal and distal position below 0.7 should be chosen to mitigate stress shielding effects, although further assessment of the clinical implications is warranted.

In our study, this overfilling of the humeral canal was avoided as described in the surgical technique. In another study [12], also with this care on the surgical technique to avoid direct contact of the prostheses with the cortical bone showed, only a 17% overall of bony adaptations were observed.

The absence of revisions for aseptic humeral loosening, consistent with prior studies [12, 36, 43], raises questions about the clinical significance of observed radiographic changes. Long-term studies are necessary to determine whether these changes are adaptive or require further intervention.

Complication and revision rates in this study align with existing literature [12, 43], with rare complications, a 2% humeral loosening rate, a 3% overall revision rate, and < 1% for aseptic humeral loosening [43]. However, the study acknowledges limitations such as non-randomized cases, absence of a control group with a conventional longer stem, and single-center radiological data review. Despite these limitations, rigorous standardization enhances result reliability.

Over the past two decades, only around 20 studies have reported on specific humeral stem designs, with most focusing on short-term outcomes in anatomical shoulder arthroplasties [43, 44]. This study contributes significantly to the knowledge on cementless short stems in RSA, particularly in an older population.

As the elderly population grows, the study anticipates an increase in revision cases. The challenges associated with longer humeral stems, including bone loss and fixation issues, underscore the importance of research in this area. Notably, short stem prostheses, as highlighted in this study, offer advantages in addressing periprosthetic fractures, particularly in the proximal region, crucial in a population predisposed to falls.

## Conclusion

In conclusion, cementless reverse short stem shoulder arthroplasty using the Aequalis Ascend™ Flex Convertible Shoulder System (Stryker®) proves to be a viable option, even for patients aged 70 years and above. This procedure offers stable fixation and produces positive clinical and radiological results during short-term follow-up.

### Electronic supplementary material

Below is the link to the electronic supplementary material.

Supplementary Material 1
