# Supplementary material for: Outcomes of a cementless onlay short stem reverse shoulder arthroplasty in elderly patients: a comprehensive analysis of clinical and radiological findings
Source: Arch Orthop Trauma Surg. 2024 Apr 23;144(5):2093–9. doi: 10.1007/s00402-024-05321-6 (PMC11093792; doi:10.1007/s00402-024-05321-6)

Conflict of interest

The authors, their immediate families, and any research foundations with which they are affiliated have not received any financial payments or other benefits from any commercial entity related to the subject of this article.

Porto, 06.01.2024

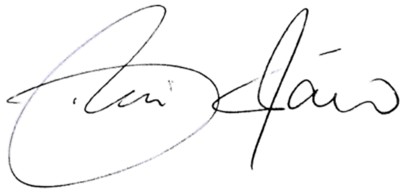

Supplement: Supplementary file 1 — Supplementary Material 1 [file 402_2024_5321_MOESM1_ESM.pdf]
